# Screening of Potential Breast Cancer Inhibitors through Molecular Docking and Molecular Dynamics Simulation

**DOI:** 10.1155/2022/3338549

**Published:** 2022-06-28

**Authors:** Sangavi Pandi, Langeswaran Kulanthaivel, Gowtham Kumar Subbaraj, Sangeetha Rajaram, Senthilkumar Subramanian

**Affiliations:** ^1^Cancer Genetics & Molecular Biology Laboratory, Department of Bioinformatics, Science Campus, Alagappa University, Karaikudi, Tamil Nadu, India; ^2^Faculty of Allied Health Sciences, Chettinad Hospital and Research Institute, Chettinad Academy of Research and Education (Deemed to Be University), Chennai, Tamil Nadu, India; ^3^Department of Physics, Mannar Thirumalai Naicker College, Pasumalai, Madurai, Tamil Nadu, India; ^4^School of Medicine, College of Medicine and Health Science, Jigjiga University, Jigjiga, Ethiopia

## Abstract

Cyclooxygenase-2 (COX-2) is a key enzyme involved in overexpression in several human cancerous diseases including breast cancer. By performing efficient virtual screening in a series of active molecules or compounds from the Maybridge, NCI (National Cancer Institute), and Enamine databases, potential identification of COX-2 inhibitors could lead to new prognostic strategies in the treatment of breast cancer. Based on a 50% structural similitude, compounds were chosen as the inductive model of COX-2 inhibitions from these databases. Selected compounds were filtered and tested with Lipinski's rule of five followed by absorption, distribution, metabolism, and excretion (ADME) properties. Subsequently, molecular docking was performed to achieve accuracy in screening and also to find an interactive mechanism between hit compounds with their respective binding sites. Simultaneously, molecular simulations of top-scored compounds were selected and coded such as Maybridge_55417, NCI_30552, and Enamine_62410. Chosen compounds were analyzed and interpreted with COX-2 affinity. Results endorsed that hydrophobic affinity and optimum hydrogen bonds were the forces driven in the interactive mechanism of *in silico* hits compounds with COX-2 and can be used as efficient alternative therapeutic agents targeting deleterious breast cancer. With these *in silico* findings, compounds identified may prevent the action of the COX-2 enzyme and thereby diminish the incidence of breast cancer.

## 1. Introduction

In current literature, breast cancer is a disease represented by a tumor microenvironment marked by inflammatory cells, involved in the neoplastic process by accelerating proliferation, withstanding and transfer of tumor cells from one part to another using receptors [1]. Breast cancer is a recurring cancer among women, influencing 2.1 million women individuals each year, and caused the maximum number of deaths among women in 2018 [2]. It was estimated that 627,000 women died from breast cancer, which accounts for 15% of all cancer-related diseases. The incidence rate of breast cancer was higher among women in developed countries and the rates were accelerating in every region of the world [3].

Recent studies depicted the components of inflammation and tumor proliferation that has been intensively studied by several researchers worldwide. Cyclooxygenase-2 (COX-2) is a predictive factor for tumor progression and associated with carcinogenesis, focusing on angiogenesis and breast cancer [4]. The physiological mechanism of COX-2 reports that prostaglandins are generated from arachidonic acid involving two isoenzymes such as COX-1 and COX-2, stimulating metabolism of arachidonic acid to prostaglandins (PG), inturn converted into prostaglandin H2 (PGH2) using glutathione-dependent peroxidase. This mechanism was shown to get involved in the progression of inflammation [5]. Prostaglandin performs its role by ligand binding with specific G-protein receptors in signal transduction stimulated angiogenesis. Therefore, COX-1 and COX-2 are involved in characterizing the inflammation process and play a key role. It is noteworthy that COX-1 exerts no significant role in promoting breast and lung cancer [6]. Published literatures established that the COX-2 expression was found high in 50% of all breast cancers, and the level of COX-2 was positively associated with tumor invasiveness [7].

A recent study reported that nonsteroidal anti-inflammatory drug (NSAID) has played an appropriate role for arresting COX enzyme activity, especially in the arachidonic acid signal pathway thereby preventing the generation of PGs and other inflammatory mediators in breast cancer cases. However, therapeutically approved and selective inhibitors for better gastric safety and breast cancer showed adverse cardiovascular side effects, leading to provoke researchers to identify and evaluate an alternate with potential COX-2 inhibitory activity [8]. Development of selective inhibitors for COX-2 targeting Parkinson's and Alzheimer's diseases is still under debate followed by cancer chemotherapy and neurological disorders. Although NSAID was administered, adverse effects were attributed to coexisting suppression of COX-1 and COX-2 enzymes that caused side effects such as renal defects and gastric ulcer [9]. Hence, developing COX-2 inhibitors with the potential of suppressing the proliferation of tumor cells and inflammation with the least adverse effects is a demanding task and highly debatable. COX-2 has a bigger (approximately 20%) and more accessible channel as a result of three amino acid changes. A structural alteration occurs when a valine at position 523 in COX-2 is replaced by a rather bulky isoleucine (Ile) residue in COX-1 at the same position in the enzyme's active region. This COX-2 enzyme alteration enables access to an extra side pocket, which is required for COX-2 drug selectivity. In the case of COX-1, access to this side pocket is restricted. Furthermore, replacing Ile-434 with a valine in COX-2 allows phenylalanine-518 (Phe-518), a nearby residue, to swing out of the way, allowing more access to the side cavity. Another critical amino acid difference exists between the two isoforms, which alters the chemical environment rather than the geometry of the drug-binding site. COX-2 has an arginine in place of histidine-513 (His-513) in the side pocket, which can interact with polar moieties. The selectivity profile of inhibitors is greatly influenced by these changes across COX active sites.

Several efforts were made to ascertain structures using common pharmacophore to arrest the COX-2 activity were attempted earlier [10, 11]. To identify selective bioactive molecules or compounds, a virtual screening technique is appropriate. Virtual screening is one of the efficient methods often used in the invention of novel drugs and is an applied strategy. In virtual screening, a big library of chemical entities has been screened to select suitable ligand-receptor interaction patterns to minimize time and money in the drug invention processes. Several virtual screening studies were employed on the docking method benefits on different databases. Novel biomolecules from the databases were influenced by physic-chemical properties. A recent study elaborately described structural resemblance for natural products in selecting anti-inflammatory inhibitors using molecular docking techniques [12]. Therefore, in the present investigation, molecular docking and molecular dynamics simulation (MDS) by using physiochemical filters were done to identify novel hit lead compounds or active molecules capable of COX-2 inhibitory effects from three chemical databases.

## 2. Materials and Methods

**Figure 1:**
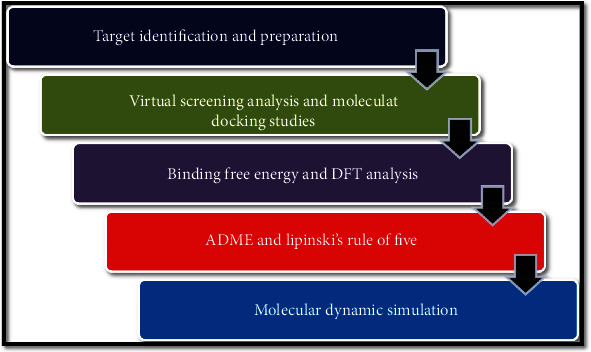
The workflow.

The overall computation techniques were carried out in Schrodinger Suite 2017 version. The workflow was depicted in Figure 1.

### 2.1. Virtual Screening

Virtual screening is a common efficient technique used in designing drugs towards the development of pharmacological central compounds by academic research groups [13]. The structure of COX-2 was recovered from the protein data bank with the identification code PDB ID-5F19 (https://www.rcsb.org). Before initiating virtual screening, it is mandatory to build a library of the promising molecule. To achieve this, a resembling model was recovered from three databases such as Maybridge (https://www.alfa.com/en/maybridge-pre-plated-screening-compounds-and-fragment-libraries/), NCI (https://cactus.nci.nih.gov/download/nci/), and Enamine (https://enamine.net/compound-collections/real-compounds/real-database) databases.

### 2.2. Lipinski's Rule of Five Parameters

To achieve good access to drugs, Lipinski's rule of five [14] was followed. It serves as a filter for virtual screening of the selected database (have a violation of about 0 and 1 was considered for this study). Lipinski's rule states that the small compounds with a molecular mass less than 500 Da, no more than 5 hydrogen bond donors, no more than 10 hydrogen bond acceptors, and an octanol–water partition coefficient log *P* not larger than 5 have traditionally been used as medicines. These are all the four rules of Lipinski's rule of five.

### 2.3. Ligand Preparation (Lig Prep)

This is a strong assembling tool that is designed with high quality with large number of drugs like molecular 2D and 3D structures that are pooled as structure data file (SDF). Force field geometry was optimized in the model, and partial atomic charges were calculated using the OPLS_2005 force field [15].

### 2.4. Grid Generation for Receptors

To generate the Grid box, receptor grid generation was done with the needed protein template along with bond order and formal changes. It involves four taps, receptor, site, constraints, and rotatable groups.

### 2.5. Glide

Glide-based ligand docking with energetics (Glide) is one of the techniques of Schrodinger Suite used for docking. Glide was used as a filter to search the ligand's location in the active site of proteins. Refinement tool Glide XP was used to dock the top-scoring ligand of the respective protein model [16].

### 2.6. Molecular Docking Protocol

Docking computation was performed using the XP mode of Glide, one of the modules of Schrodinger (2017), a ligand docking tool comprising five taps. In Glide, docking flexibility option was used to generate confirmation during the docking process. Docking scores were visualized by Glide XP visualization and provided 3D visualization for the XP term. Energy evaluation was carried out using glide score, and the right pose was determined from the output to the respective ligand [17, 18].

### 2.7. Binding Free Energy

Optimized potential for ligand stimulation (OPLS-AA) 2005 force field and GB/SA solvent modeling was performed in the process of calculating the binding free energy [19].

### 2.8. Density Functional Theory (DFT)

Jaguar module was used to compute DFT in our study. Calculation of the highest occupied molecular orbital (HOMO) and the lowest unoccupied molecular orbital (LUMO) was done. To mimic physiological conditions, energy calculation was assumed. Quantum chemical descriptors covering MESP, HOMO, and LUMO ,and aqueous solvation energy were employed and computed. Following equation was used to derive the density (*r*):

(1) $$\begin{array}{c} V\left(r\right)=\overset{N}{\underset{A-1}{\sum }}\left(\frac{ZA}{r-R}-\frac{p\left(r^{\prime }\right)d^{3}r^{\prime }}{\;|\;r-r^{\prime }\;|\;}\right). \end{array}$$

Here, *N* is the total number of nuclei in the molecule and the two terms refer to the bare nuclear potential and the electronic contributions, respectively [20]. Jaguar (v8.7) module was used to calculate the molecular electrostatic properties, namely, MESP, HOMO, LUMO, and dipole movement. After the completion of calculation of electrostatic potential energies, negative and positive regions of the compounds were indicated by different color variations [21, 22].

### 2.9. ADME Prediction

Efficiency and safety of compounds are important factors to expose in the market and such factors could be tested using ADME and toxicity profile [18, 23–25]. Based on pharmaceutical relevance criteria, selected compounds were considered a drug-like molecule, in contrary to the compounds with unsuitable properties elevate the drug development cost and cause burden to patients [13]. Therefore, Qikprop module was used to assume the ADME properties of the screened compounds.

### 2.10. Molecular Dynamic Simulation

GROMACS (http://www.mdtutorials.com/gmx/complex/index.html) was used to analyze the molecular dynamics simulation. To check the stability of the protein-ligand complexes in contrast to molecular docking, water molecules were the one among important factors to be considered in molecular dynamics simulations, which reveals better binding confirmations for the docked complex and closely acknowledge the physiological environment conditions. Selection of complexes for molecular dynamics simulations were based on the above docking results. RMSD of the protein backbone was analyzed for each complex to evaluate the stability of the protein and their confirmation changes during 50 ns. Understanding the interaction of docked complexes during 50 ns periods was mandatory to understand its inhibitory mechanisms by analyzing root mean square fluctuation (RMSF) for the complexes that were identified. Of all the compounds, the best three from these databases, which were interacting with the active site of COX-2 protein has been chosen for molecular dynamic simulation studies at 50 ns. RMSD value has been used to analyze the stability of a protein. Analysis of the dynamic behavior of the protein-ligand complex using force field [19] and its structural coordinates was done using analytical tool in the GROMACS 4.6.1 package [17, 26, 27].

## 3. Results and Discussion

### 3.1. Virtual Screening of COX-2 Protein

Identification of novel inhibitor compounds against the COX-2 protein was done using hierarchical complex screening method. As the first step, the National Cancer Institute (NCI), Maybridge, and Enamine databases were screened. Such compounds were screened carefully, and the crystallized ligand residue was observed and its active sites were found to be H_207 and N_382. Such screened compounds were further subjected to the analysis. Results of NCI, Maybridge, and Enamine database compounds exhibited the highest docking score and were recorded -8.859 kcal/Mol, -10.503 kcal/Mol, and -8.584 kcal/Mol, respectively.

### 3.2. Protein-Ligand Docking

**Figure 2:**
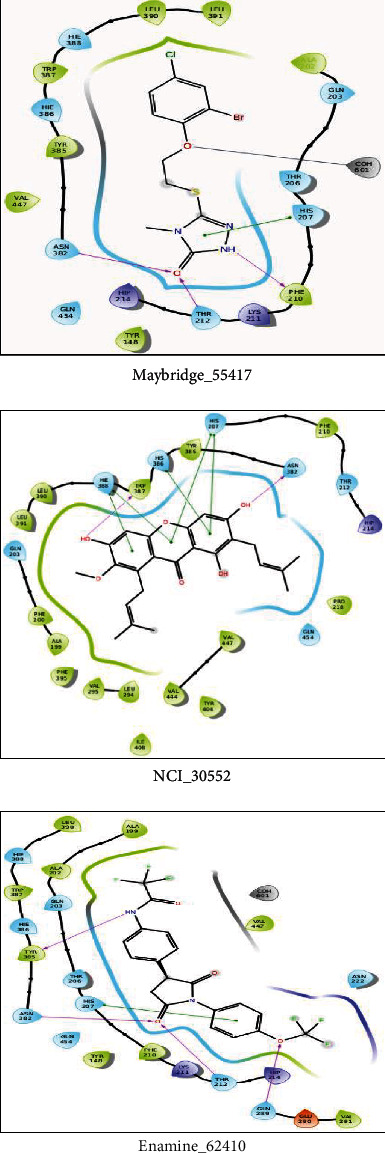
Protein-ligand interactions of selected compounds of the Maybridge, NCI, and Enamine databases with best binding poses and interactions of compounds in the active site of protein.

The cocrystallized ligand was redocked with the target protein (COX-2) for analyzing the interacting residues of the lead compounds, when docked with the target protein. The interacting pose was depicted in supplementary figure 1. This was performed by applying glide application docking precision for the chosen 3 compounds based on the criteria of glide XP score, number of interactions, and Glide energy computation (Table 1). The size of grid box was *X* = 20.72, *Y* = 37.54, *Z* = 59.43. Best 3 identified leads were encoded as Maybridge_55417, NCI_30552, and Enamine_62410. In the view of the hydrogen bond, interaction with their respective residue in the NCI database was found to be H_207, H_386, and HIE_388 residues, whereas Pi-Pi interaction was shown with W_387 and N_382 residues. Consequently, in the Maybridge database, one compound appeared to have a high docking score with hydrogen bond interaction in H_207 residue, whereas Pi-Pi was stacked with F_210, T_212, and N_382 residues. Enamine database showed a hydrogen bond interaction with H_207 residue while Pi-Pi interaction with Q_289, T_212, Y_385, and N_382 residues (Figure 2). The major hydrophobic channel, the catalytic Ser-530, and the mouth with polar residues such as Arg-120 are all shared by COX-1 and COX-2 active sites. COX-1, on the other hand, lacks the side pocket and has a narrower main hydrophobic channel.

### 3.3. Free Energy Computation (Prime MM-GBSA)

Free energy binding of selected compounds with their respective receptors was computed by Prime MM GBSA (Molecular Mechanics Generalized Born Surface Area) approach. Free energy of identified inhibitors was found to be -59.958, -44.559, and -52.341 kcal/mol by their respective encoded compounds in Maybridge, NCI, and Enamine databases, respectively. Lowest binding energy referred the best binding affinity of the complex. The relative binding energies of complexes are described using the prime energy calculation technique. It was discovered that binding of both ligands to the COX-2 protein is a thermodynamically advantageous process.

### 3.4. Density Theory Calculation

**Figure 3:**
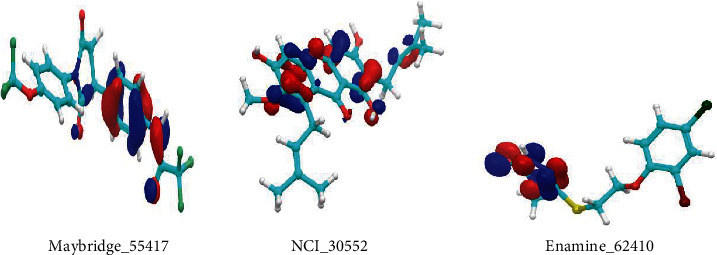
HOMO of lead potential compounds.

**Figure 4:**
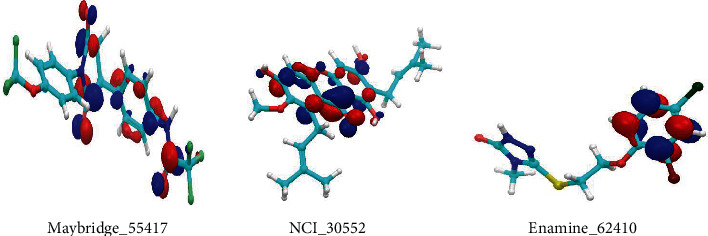
LUMO of lead potential compounds.

**Figure 5:**
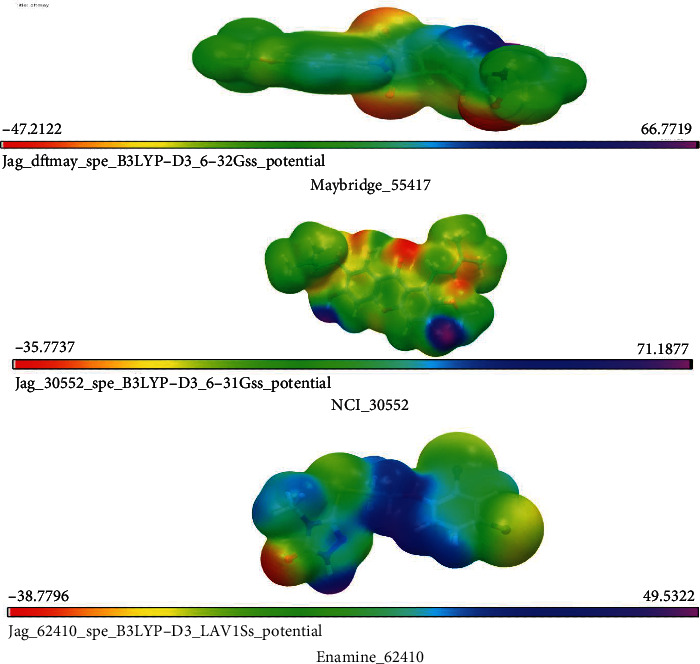
MESP of lead potential compounds.

Three screened compounds were analyzed using distribution of frontier molecular orbital measure that represented nucleophilic and electrophilic attraction in charge transfer reaction. However, the energy gap between the HOMO and LUMO generated an unsuited state of electron transition and declined the reactive ability (Table 2). And the poses of potential lead compounds of HOMO (Figure 3), LUMO (Figure 4), and MESP (Figure 5) were displayed.

### 3.5. Association of COX-2 with Breast Cancer

Association of COX-2 inhibitors such as celecoxib and nimesulide with breast cancer was studied by many research groups in mouse models indicated that inhibition of COX-2 delayed the onset of breast tumor formation and declined disease occurrence [28]. A study report has demonstrated that overexpression of COX-2 was adequate to stimulate carcinogenesis in HER2-neu (human epidermal growth factor receptor) mice after multiple gestations. These findings exhibited the crucial role of COX-2 inhibitors influenced by breast cancer development [29]. COX-2 was shown too involved in the adaptative cytoprotection response in gastrointestinal (GI) mucosa. Inflammation or ulceration in the GI mucosa could rapidly induce COX-2 at sites of injury where it produces large amounts of PGs involved in the healing process [30]. Perhaps, avoidance of COX-2 inhibitors in patients with gastric susceptibility could help better prognosis. Therefore, in the present investigation, a virtual screening technique was employed and 3 potential leads novel molecules were identified that could minimize the risk of breast cancer. The RMSD plot showed that NCI_30552 compound has good stability from 20 to 50 ns. In general, the higher the fluctuations found at the end of the plots correspond to the termini. These results indicated that the fluctuation rate of the protein-ligand complex was excellent in 400-500 residues and has been concluded that these fluctuations do not affect the overall structural stability of the protein at the active site.

### 3.6. ADME Property Prediction

ADME predictions were done to test drug-likeness and pharmaceutical characteristics of identified lead compounds for chances of success in pharmaceutical industry. By achieving the filtration process, chosen compounds were passed to the next step of Lipinski's rules of five and ADME properties analysis. Absorption, distribution, metabolism, excretion, and toxicity (ADMET) qualities must be predicted in silico in order to pick the most promising compounds for further research. The pharmacokinetic parameters of the chosen structures were predicted, including absorption, distribution, metabolism, and elimination. These results were tabulated in Table 3. In the Qikprop module, ADME properties of identified compounds explored were highly suited for drug formulation and these compounds obey Lipinski's rule of five together with other pharmaceutical relevant parameters like molecular weight, human gastrointestinal absorption, and blood-brain barrier.

### 3.7. MDS Studies on Potential Lead Compounds with COX-2 Protein

**Figure 6:**
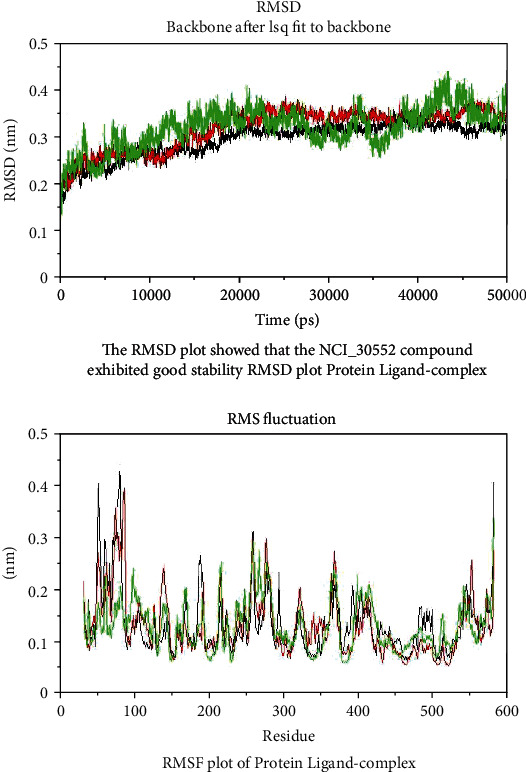
Time dependence of the radius of gyration (Rg) graph of Maybridge_55417 NCI_30552 and Enamine_62410 complexes. Red color for Maybridge_55417 compound, black color indicates NCI_30552 compound, and green color indicates Enamine_62410 compound at different time scales (20 to 50 ns).

**Figure 7:**
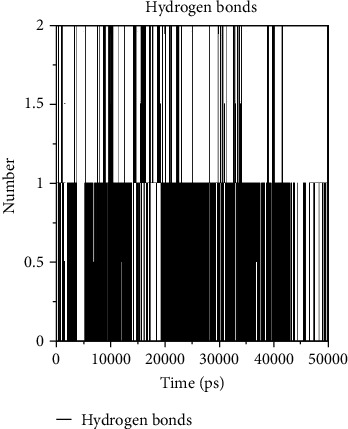
Hydrogen bond interaction between protein-ligand complexes.

Stability of the protein-ligand complex was studied by using molecular dynamic simulation. Each system was subjected to position restrained simulation for 50 ns, and the RMSD (root mean square deviation) was calculated concerning the starting structure as a function of time to evaluate the degree of conformation of the protein. From Figure 6, the RMSD plot showed that the NCI_30552 (black color) compound has good stability from 20 to 50 ns. Then, the Maybridge_55417 (red color) compound and Enamine_62410 (green color) compound have moderate deviations and have less stability throughout the simulation period. During the period of simulation, RMSF was used to estimate the average fluctuations of the macromolecular target protein at the residue level and peaks indicate the most fluctuating protein. In general, higher fluctuations found at the ends of the plots correspond to the termini. These results indicated that the fluctuation rate of the protein-ligand complex was good from 400-500 residues (Figure 6). These protein-ligand interactions revealed the information of active site residues and part of ligand that influence the binding affinity. However, these fluctuations do not affect the overall structural stability of the protein at the active site. Monitoring of time dependence of hydrogen bonds between the receptor and the small molecule could help to understand the binding nature of lead molecules in the active site of COX-2, which showed very well correlations between the biological activities of the small molecules and the calculated intermolecular hydrogen bonds. In our study, a direct clue about the affinity of the identified lead molecule towards the protein-ligand complex has been shown with evidence of constant hydrogen bond interaction throughout the simulation period (Figure 7).

## 4. Conclusion

In the present study, protein-ligand interaction was studied using docking, compound stability, and hydrogen bond interaction by employing molecular dynamics simulation from the Maybridge database, NCI database, and Enamine database. Highest docking score was shown by NCI_30552 and Maybridge_55417 compounds with -10.503 and -8.859 kcal/mol free energy, respectively. RMSD results showed that NCI_30552 exhibited good protein-ligand interaction and stability compared with others. Taking into account of these results, it was inferred that three hit compounds that were screened have good docking scores and showed a greater affinity with target COX-2 protein. In conclusion, the identified compounds might be useful for further studies leading to clinical trials towards inhibitory activity of the COX-2 enzyme, thereby minimizing the occurrence of breast cancer.

## Figures and Tables

**Table 1:** Illustrates the selected potent compounds and their Glide XP, fitness scores, and their interaction residues with COX-2 pattern.

S. no	Compound ID	Glide XP score (kcal/Mol)	Glide energy (kcal/Mol)	Interacting residue	Fit score
1	Maybridge_55417	-10.503	-59.842	H_207 (HB) F_210, T_212, N_382 (PI-PI)	1.239
2	NCI_30552	-8.859	-52.268	H_207,H_386, HIE_388 (HB)W_387, N_382 (PI-PI)	1.564
3	Enamine_62410	-8.584	-45.301	H_207(HB) Q_289, T_212, Y_385, N_382 (PI-PI)	1.673

**Table 2:** Showing binding free energy scores and HOMO, LUMO, and MESP of hit potential compounds.

S.no	Compound ID	Δ*G* bind (kcal/Mol)	HOMO (eV)	LUMO (eV)	Solv. energy (kcal/Mol)
1.	Maybridge_55417	-59.958	-0.23966	-0.04770	-18.26
2.	NCI_30552	-44.559	-0.21548	-0.06670	-14.51
3.	Enamine_62410	-52.341	-0.13743	-0.03969	-8.88

**Table 3:** Showing ADME properties of identified compounds using the Qikprop module.

S.no	Compound ID	MW	Donor HB	Accept HB	%human oral absorption	QPlogPo/w	QPPCaco	QPPMDCK	Rule of five
1.	Maybridge_55417	446.31	1.000	5.500	100	4.723	693.684	7564.41	0
2.	NCI_30552	410.42	2.000	4.500	100	4.852	584.333	276.782	0
3.	Enamine_62410	263.21	3.000	5.250	100	1.027	317.25	3089.67	0

## Data Availability

Sufficient data have been included in the manuscript. Additional data can be kindly requested from the corresponding author.

## Abbreviations

ADMET: Absorption distribution, metabolism, excretion, toxicity
COX-2: Cyclooxygenase-2
DFT: Density functional theory
GROMACS: GROningen MAchine for Chemical Simulations
HER2: Human epidermal growth factor receptor 2
HOMO: High occupied molecular orbital
LUMO: Lowest unoccupied molecular orbital
MDS: Molecular dynamics simulation
MESP: Molecular electrostatic potential
MMGBSA: Molecular mechanics generalized bond surface area
NCI: National Cancer Institute
NSAID: Nonsteroidal anti-inflammatory drugs
OPLS-AA: Optimized potential for liquid simulation-all atom
PG: Prostaglandins
PGH2: Prostaglandins H2
RMSD: Root mean square deviation
RMSF: Root mean square fluctuation
SDF: Structure data file
SP: Standard precision
XP: Xtra Precision.

## Supplementary Materials

Supplementary Materials Supplementary Figure 1: COX-2 with the cocrystalized ligand.
