# Supplementary material for: Screening of Potential Breast Cancer Inhibitors through Molecular Docking and Molecular Dynamics Simulation
Source: Biomed Res Int. 2022 Jun 28;2022:3338549. doi: 10.1155/2022/3338549 (PMC9256436; doi:10.1155/2022/3338549)
Supplement: Supplementary Materials — Supplementary Figure 1: COX-2 with the cocrystalized ligand. [file 3338549.f1.zip › Supple.Description.docx]

**Supplementary Figure 1: COX-2 with the co-crystalized ligand**
