# Supplementary figures and images for: Screening of Potential Breast Cancer Inhibitors through Molecular Docking and Molecular Dynamics Simulation
Source: Biomed Res Int. 2022 Jun 28;2022:3338549. doi: 10.1155/2022/3338549 (PMC9256436; doi:10.1155/2022/3338549)

**Supplementary Figure 1:**

**COX-2 with the co-crystalized ligand**

**
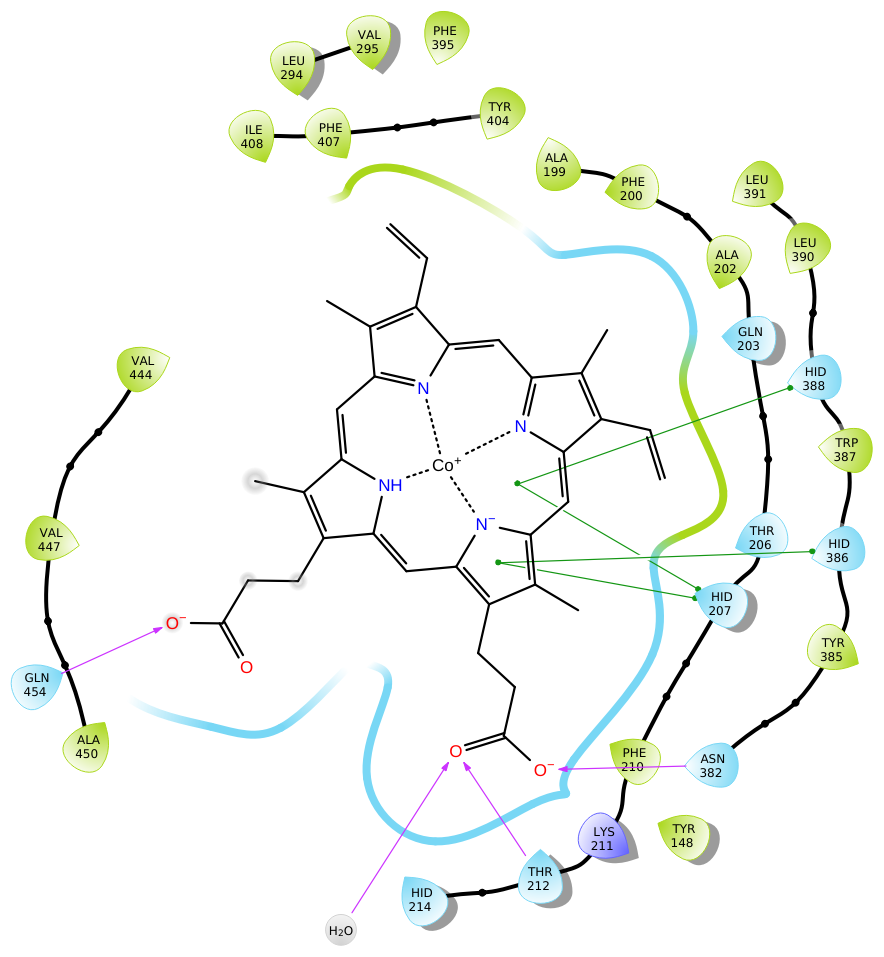
**

Supplement: Supplementary Materials — Supplementary Figure 1: COX-2 with the cocrystalized ligand. [file 3338549.f1.zip › Suppl figure 1.docx]
